# Pathology and Practice of Medicine

**Published:** 1860-01

**Authors:** Richard J. Dunglison


### PATHOLOGY AND PRACTICE OF MEDICINE.

BY RICHARD J. DUNGLISON, M.D.

I.—*Movable Kidney, in connection with Spinal Disease.* By WILLIAM HENDERSON, M.D.

About thirty cases of movable kidney, in connection with spinal disease, have already been recorded, and Dr. Henderson, Physician to the Clifton (England) Dispensary, publishes another, which has several points of interest. The patient, a female, fifty-one years of age, presented a tumor in the right hypochondriac region, which could be moved toward the umbilicus, and which disappeared under the liver when slight pressure was applied. The swelling was not painful, and resembled a healthy kidney in form and dimensions. The amount of urine passed was normal, and unaffected by pressure. While suffering from an attack of pleuro-pneumonia, she experienced severe pain in the region of the fifth and sixth dorsal vertebræ, which had been diseased for four years or more previously. During the attack she several times expectorated fetid pus in considerable quantities, and her countenance finally presented the appearance of suffering from malignant disease. Under a second severe attack of pleuro-pneumonia she died, about fifteen months after the tumor had been first examined. Immediately before her death she felt a sensation as if something had burst in her abdomen. Dr. Henderson made a post-mortem examination, and discovered extensive disease of some of the spinal vertebræ, while the right kidney was found to be perfectly movable, and suspended by a prolongation of the peritoneum inclosing the kidney. Dr. Henderson regards it as a matter of surprise that paralysis did not exist in connection with such extensive disorganization of the vertebral bones, the bodies of the fifth and sixth vertebræ being gone almost entirely, an open space being left large enough for two fingers to pass into the spinal canal. The position and relation of the kidney were no doubt congenital. No pathological condition was found to account for the sensation experienced just before dissolution.—(*Medical Times and Gazette*, November 19, 1859.)

II.—*Albuminuria in one Kidney, Saccharine Urine in the other.* By DR. GIBB.

Many interesting cases of disease of the kidney and supra-renal capsule have lately been described in the foreign and American medical journals. Some of these have presented but few points of interest, apart from the natural history of disease of those organs, as usually detailed. In a specimen exhibited before the Pathological Society of London, by Dr. Gibbs, it was conclusively shown, by the usual chemical and other tests, that one kidney secreted saccharine urine, while that from the other kidney was albuminous, the specific gravity and general characters of each varying greatly. The right kidney weighed seven ounces and three-quarters; the left weighed three ounces; the former containing two cancerous tubercles in its structure; the substance of the smaller kidney having been almost entirely absorbed. Cancer and tubercle existed in the lungs, and almost all the viscera were found to be affected with cancerous deposits.—(*London Lancet*, November, 12, 1859.)

III.—*Fatal Case of Contracted Bright's Kidney, with Pericarditis, etc.* By George Johnson, M.D.

In a case of pericarditis, which presented itself in the wards of King's College Hospital, London, examination was made by Dr. George Johnson, the attending physician, to discover, if possible, whether any renal disease had preceded the cardiac affection. Dr. Johnson made some remarks on the importance of tracing the connection existing between morbid conditions of the abdominal and thoracic viscera, and a knowledge of these mutual relations had induced him, in this case, to look to the condition of the kidneys. The urine was found to be pale, of a specific gravity of 1·011, containing albumen, and was sometimes passed involuntarily. It occasionally happens in bronchitic affections, that the urine becomes slightly albuminous, on account of a congested state of the circulation from obstruction in the chest; but Dr. Johnson inferred, in the present case, that such a condition did not satisfactorily account for it, inasmuch as albuminous urine from pulmonary disease is usually high colored, and is generally accompanied with copious deposits of lithates, characteristics which were absent in the pale urine examined. He diagnosticated, therefore, the existence of Bright's disease of the kidney, the organs being in an advanced condition of degeneration. He considered that the bronchitis and pericarditis existing had been caused by contamination of the blood with urine. The remedies adopted —cathartics, and a stimulating regimen—were unsuccessful in arresting the progress of the disease. No dropsical effusions accompanied it. Dr. Johnson regards the microscopic examination of the urine as a very important means of diagnosis, the tube-casts affording information, especially when albumen may not be detected in the urine by other tests.—(*Medical Times and Gazette*, October 15, 1859.)

IV. *Total Suppression of Urine.* By Mr. J. B. Jeaffreson.

A case is reported by Mr. Jeaffreson, of Monmouthshire, England, of an Irish woman, thirty-eight years of age, who, after giving birth to a still-born child of about eight months, was attacked with violent pain in the abdomen and left lumbar region, the abdomen being considerably distended. On the second day after her confinement she passed no urine, and but half a drachm came away when the catheter was introduced. She was put upon the use of dilute hydrocyanic acid, sweet spirits of nitre, and nitrate of potassa, while opium, calomel, and camphor were also prescribed. Three days afterwards, not having passed any urine since the catheter was employed, the instrument was again introduced; but not a drop of urine was passed. This condition continued for seven days, the urine being totally suppressed, and the usual diuretic treatment failing to have any effect. The patient survived about two days longer than any of the previously recorded cases of the same kind, and without any symptoms of uræmic poisoning.—(*London Lancet*, New York edition, December, 1859.)

V.—*Interesting Cardiac Affections. Rupture of the Aortic Valves.* By DR. SANDS.—*Rupture of the Chordæ Tendineæ.* By DR. DALTON.

In the Proceedings of the New York Pathological Society, for September fourteenth, we find two interesting illustrations of cardiac disease, in one of which the aortic valves were ruptured, and in the other the chordæ tendineæ attached to the anterior fleshy columns of the heart.

Dr. Sands presented to the Society a heart, which had been removed from the body of a policeman, who had received injuries while in the discharge of his duties, which had, probably, some connection with his death. Two days after the capture of his prisoner, he complained of pain in his chest; but no other symptoms supervened for ten days, at the expiration of which time there were signs of pleuritic inflammation, and a systolic murmur was heard in the region of the base of the heart. The pulse increased from 80 beats to 140 or 150, and then became almost too rapid to be counted. Dyspnœa accompanied these symptoms. On post-mortem examination, the chief point to be noticed was a lesion of the aortic valves, but one of them remaining intact. Of the other two valves, one is almost entirely gone; the other has a large rent, extending nearly the whole length of the valve; while the valve opposite the right coronary artery has a fibrous deposit upon it, and the endocardial lining membrane torn. The great difficulty, in such cases as Dr. Sands has described, is in diagnosticating the precise morbid condition that has supervened. Even violent external injury would not prepare us to find such changes in the important central organ of the circulation as were found here. It would be an interesting point to know, if the patient had previously suffered from cardiac disease.

Dr. Dalton described a case of rupture of the chordæ tendineæ, where the only symptoms presented were throbbing of the carotids, a bellows murmur at the præcordium, loudest at the base of the heart. The patient was unconscious, and his breathing was stertorous. Death took place three hours after the attack. No clots or fibrinous deposits were found in the heart, and no lesion except rupture of the chordæ tendineæ.

Cases of this kind are more interesting as pathological specimens than as means of instruction in therapeutics. The difficulty of diagnosis, and the impossibility of rendering any medical assistance, even if it were possible to make a clear diagnosis, do not render them of any striking value to medical practitioners. They belong to the *res curiosæ* of our profession, which deserve to be recorded, but which lead to but little practical benefit in the therapeutics of cardiac diseases.

VI.—*Rupture of the Right Auricle of the Heart.* By J. N. OREGEN, M.R.C.S.

A case, in some respects similar to those previously recorded, has been described by Mr. Oregen, a post-mortem examination having been made on the body of a man who had died suddenly. He had met, two or three months previously, with an accident, by the falling of a heavy piece of iron on his head; and the post-mortem examination was made with a view of discovering whether any lesions existed sufficient to account for this accident as a cause of death. No fracture was discovered, and the brain itself was healthy. On examining the chest, the pericardium was found distended with a very large quantity of semi-coagulated blood. The right auricle was found to have a rent situated between the entrance of the inferior vena cava and the right auriculo-ventricular opening, about six lines in length, and partly filled with a plug of fibrin. No dilatation or fatty degeneration of the right auricle existed, and the valves were perfectly healthy.

Mr. Oregen speaks of the rarity of these cases of rupture of the heart. By a singular coincidence, the descriptions of three interesting examples are contained in the journals. Violent mental emotion, the usual cause assigned for such a condition, did not exist in this case.

Professor Taylor, in his work on Medical Jurisprudence, mentions the case of a young man, somewhat similar in its history to the above, although the patient survived ten hours after his seizure, while in Mr. Oregen's case the patient survived but eight minutes.—(*London Lancet*, New York edition, December, 1859.)

VII.—*A Case of Aneurism of the Abdominal Aorta arrested in its Progress.* By SAMUEL SOLLY, F.R.S.

The Proceedings of the Royal Medical and Chirurgical Society for November 8, 1859, contain an interesting discussion on a case of aneurism, reported and very fully described by Mr. Solly.

Captain W., thirty years of age, was found suffering from intense agony, the seat of which he referred to the epigastric region of the abdomen. He could not lie flat on his back without adding to the severity of his pain. The patient complaining of constipation, free motion of the bowels was produced by croton oil, and as soon as they had acted freely, a grain of morphia, with three drops of tincture of aconite, was given every three hours. It was difficult to decide what was the cause of the intense pain, until the history of the case showed that four years previously he had suffered from similar symptoms while actively engaged on horseback in following the hounds. The pain continued some hours afterwards, and he was never perfectly well up to the time of Mr. Solly's first visit.

Upon a careful examination, a distinct pulsating tumor was found a little above the umbilicus, at about the bifurcation of the aorta. The pulsation was felt not merely from behind forward, but laterally also. A distinct *bruit de soufflet* was heard upon auscultation, and the diagnosis that it was an abdominal aneurism was concurred in by Dr. Watson, who saw the case in consultation with Mr. Solly. This diagnosis was also confirmed by Mr. Leggatt, who made a record of the daily changes in the patient's condition. The pain disappeared temporarily under the anæsthetic influence of chloroform, but on its recurrence a drachm of tincture of opium was given him by injection. Sir Benjamin Brodie was also called to see the case. Mr. Leggatt could not discover, five days after Mr. Solly first saw the patient, any pulsation in the right iliac artery, and Sir B. Brodie and Mr. Solly could not as it passed over the pubis. In the left iliac and femoral arteries pulsation was distinct but feeble.

Thirteen days after Mr. Solly's first visit the aneurism was discovered to be smaller, the pulsation less distinct, and no *bruit* perceptible. He was ordered an aromatic mixture of the disulphate of quinia, sulphuric acid, alum, etc., which was abandoned soon, and quinia and creosote given to allay the sickness which had been persistent. The pulse soon became stronger, and a slight *bruit* became audible in the aneurismal tumor. The aneurismal thrill and pulsation and the whirring sound became again very distinct, and a whistling sound also accompanied it. A recumbent posture was recommended and entire confinement to bed. The tumor continued to grow larger, and he was placed upon the starving system. Six months after Mr. Solly's attention was called to him, the pulsation was found to be more distinct, and to extend more in the direction of the right groin. In less than a month after this, word was sent from Dover, to which place Captain W. had been removed, that "nature was performing a cure which no operation could have done;" pulsation became greatly diminished. Shortly afterwards, while taking exercise, the pain and nausea returned in all their intensity, and the history of the case varied but little, in the transition from intense agony to subdued pain, from that of months previously, and the patient at last died suddenly, after a very severe paroxysm, about three years after Mr. Solly ceased to see him.

Post-mortem examination revealed the cause of death to be the bursting of an aneurism of the abdominal aorta, immediately above its bifurcation, nearly four inches in length, and between two and three in breadth, the rupture occurring just above the origin of the left iliac artery. The coats of the aneurismal tumor were found plated with ossific and fibrinous deposits. These deposits, by weakening the coats, are often important causes of aneurismal tumors.

The history and treatment of this case, as detailed by Mr. Solly, gave rise to a very interesting discussion. In three cases described by Mr. Adams, an abdominal aneurism had found its way into the thorax, and burst into the left pleura. The merits of Valsalva's method of curing aneurism were canvassed, and Mr. Holmes Coote asserted that in no case had he seen it successful. Dr. Copland recollected a case of extensive aneurism of the aorta that was materially improved by rest, anodynes, a regulated diet, and attention to the normal condition of the bowels.—(*London Lancet*, Nov. 26, 1859.)

### VIII.—*Transitory Homicidal Mania—Where does Reason end or Mania begin?* By A. DEVERGIE, M.D.

An interesting paper was read before the Imperial Academy of Medicine at Paris, on the subject of Homicidal Mania. The treatment of insanity which assumes this form is not alone interesting to those who have the care of institutions devoted to this class of affections. Questions of importance to the pathologist arise in all such cases. At what precise point does reason end; and where does insanity take its commencement? M. Devergie describes the case of a young man, nineteen years of age, son of one of the principal merchants of Bordeaux, who, on the 10th of November, 1854, dined with his father, to whom he bore very affectionate filial relations, and his stepmother, for whom he had gradually acquired dislike and intense aversion. At dessert, the young man quitted the table, and went to the drawing-room to warm himself. He then ascended to his bedchamber, took his fowling-piece and straw hat for the purpuse of taking a stroll in the open air, when an idea of suicide, which he had entertained for a month, suddenly came into his mind, and changed instantaneously into the thought of destroying the life of his stepmother. He took two of his brother's pistols from his room, although he had his own pistols already loaded, went back to the dining-room, and discharged the contents of one barrel at his stepmother. His father rose to seize him, but he fled from the house, crying, "I am a madman—an insensate! I have killed my stepmother!" surrendered himself to the police, and related circumstantially the whole occurrence.

This case is an interesting illustration of the rapid transition from reason to insanity, and from insanity to reason again. The jury acquitted him on the ground of temporary insanity.

Two or three years later, he committed suicide on the grave of his stepmother, whom he had murdered, having written in his note-book the following entry: "I wish to die upon the tomb of her whom I have so much loved and regretted." At what period did reason die out or mania begin? Was he rational during the whole period from the murder to his own suicide?—(*Journal of Psycholog. Med. and Mental Pathol.*, October, 1859.)

IX.—*Rapidly Fatal Anœmia, with Tertian Syncopal Attacks, Purpurous Spots*, etc. By THOS. B. PEACOCK, M.D., Assistant Physician to St. Thomas's Hospital.

In a paper read before the Hunterian Society, on the subject of prevalent malarious affections, Dr. Peacock has given a statistical record of various forms of intermittent and remittent fevers, classifying them according to sex, age, etc., and cited illustrative cases as types of interesting exceptional forms of malarious diseases. One to which we would particularly refer is a fatal case of anæmia, resulting from residence in a very damp and unhealthy house. When first seen, the patient, a female, seemed to be suffering from hepatic derangement. The urine was scanty, high colored, loaded with lithates, and contained no albumen The day before Dr. Peacock saw her, as well as the day after, she was seized with a fit, which was followed by great prostration. She resided in a house situated on the side of a high hill, but the water of a well in the cellar percolated through the brick-work, so that the house generally became very damp, and consequently an unhealthy place of residence. There was no rigor attending the syncope, and great heat and fever followed the attacks. The origin of her affection being deemed malarious, she was ordered quinia and bark, with stimulants, if there should be any recurrence of them She had two syncopal attacks every other day, similar to those she had suffered from previously. Petechial spots made their appearance on all parts of the body, and a very distinct systolic murmur became audible at the base of the heart. She died from rapid exhaustion, and no post-mortem examination was made.

Dr. Peacock prefaces the report of this case with the remark, that such obscure relations of cause and effect may render it a matter of doubt whether the cause assigned be the true one. In the absence of any other apparent antecedent, malarious poison is too often assigned to justify treatment which might otherwise be considered injudicious, or to shield the ignorance of the practitioner. We do not apply, however, this remark to the case of Dr. Peacock, which seems to belong to an obscure form of malarious disease, running into a typhous condition, or, perhaps, typhous in its nature from its very inception. Exposed, however, as she was by long residence to some of the phenomena of malarious poison, it is probable that the disease remained latent, until other causes cooperated to produce a violent explosion.—(*Medical Times and Gazette*, Nov. 5, 1859.)

X. *Appendix Cœci discharged from the Bowels; Recovery of the Patient.* By Dr. Jackson.

In the Proceedings of the Boston Society for Medical Improvement for July fifteenth, may be found an interesting example of what must be an exceedingly rare affection. A robust farmer, aged twenty-four, had suffered from nausea and vomiting, distention and tenderness of the abdomen. Fears being entertained that peritonitis might occur, he was bled to the extent of a pint, but with little or no relief following. Calomel and opium, and a blister, were ordered; also a mild cathartic treatment. No movement of the bowels followed this mode of management, nor was the introduction of a tube as high up in the bowel as it could be carried attended with any better success. An infusion of tobacco (℈iv to a pint of water) thrown into the rectum, on the next morning, was followed by very great prostration, and by one or two small evacuations of a greenish mucus. On the next visit, Dr. Robbins, his attending physician, discovered an oblong tumor extending from above the spine of the right ilium nearly to the groin, to which a blister was directed to be applied. That evening, and for a day or two afterwards, the fæcal evacuations became very numerous, and the tumor gradually diminished in size. About eight days after the first appearance of the tumor, and four days after the subsidence of the symptoms had led Dr. Robbins to discontinue his visits, a substance was passed which proved, on examination, to be the appendix cæci, in a fetid, gangrenous condition. Three years had elapsed since this portion of intestine was passed, and no symptoms of inconvenience or pain had followed.

In the discussion which this case elicited in the society before which it was brought by Dr. Jackson, the hypothesis was advanced that some foreign body had probably become impacted in the entrance of the appendix, causing inflammation, obstruction of circulation, and ulceration, and finally a sloughing off of the affected portion. Dr. Jackson had never found, where the appendix was perforated, that the foreign body was at the entrance of the appendix, but midway. To express our ignorance of the functions of the appendix vermiformis cæci, it is scarcely necessary to do more than ask the question, what can be its use in digestion? It has been so often found wanting or obliterated in many subjects, that it is scarcely worthy of consideration, except as an illustration of a correspondence in structure between man and inferior animals.—(*Boston Med. and Surg. Journal*, September 15, 1859.)

